# Assembly and analysis of the complete mitochondrial genome of *Prunus davidiana* (Rosaceae)

**DOI:** 10.3389/fpls.2025.1558619

**Published:** 2025-03-27

**Authors:** Yuyan Zhang, Xin Guo, Ruijuan Ma, Mingliang Yu, Jianlan Xu, Zhijun Shen

**Affiliations:** ^1^ Institute of Pomology, Jiangsu Academy of Agricultural Sciences/Jiangsu Key Laboratory of Horticultural Crop Genetic Improvement, Nanjing, Jiangsu, China; ^2^ College of Horticulture, Nanjing Agricultural University, Nanjing, China

**Keywords:** *Prunus davidiana*, mitochondrial genome, repeats, phylogenetic analysis, indel marker

## Abstract

*Prunus davidiana* is an excellent woody species with high resistance to various abiotic and biotic stresses. The species is also a potential gene donor due to its important developmental prospects. However, the characterisation of the mitochondrial genome (mitogenome) remains unexplored. In this study, we sequenced and assembled the mitogenome of *P. davidiana* using reads from Illumina sequencing and the Oxford Nanopore platform. According to the results, the *P. davidiana* mitogenome is 407,608 bp in length, with a GC content of 45.5%. It contains 64 genes, including 39 protein-coding genes, 22 tRNA genes and 3 rRNA genes. Codon usage, repetitive sequences, nonsynonymous to synonymous substitution ratios, RNA editing, synteny, and phylogenetic relationships among Rosaceae species and gene migration events were investigated. In addition, two novel indel markers were developed to distinguish several *Prunus* species (i.e. *P. pseudocerasus*, *P. armeniaca*, *P. salicina* and *P. davidiana*). The results of our analyses provide valuable information and a theoretical basis for future research on *P. davidiana*.

## Background

*Prunus davidiana* is a small shrub belonging to the subfamily Prunoideae of the family Rosaceae (Liu et al., 2017). The species is native to China and is reported to be resistant to cold, saline alkalinity and drought stress, as well as some pests and diseases (Hesse, 1975). Therefore, it is widely used as a grafting rootstock for peach and plum in northern China due to its adaptability to different environmental conditions (Xu et al., 2020). Accordingly, *P. davidiana* is considered as a potential gene donor in future peach breeding programmes.

As the ‘energy factories’ of cells, mitochondria are important for energy synthesis and conversion in cell physiological activities (Ye et al., 2017). They play a critical role in plant growth and development (Ogihara et al., 2005). Plant organelle genomes have genetic systems that are independent of the nuclear genome and share stable regulatory mechanisms with the nuclear genome (Li et al., 2022). The chloroplast (cp) genome is usually structurally conserved with double-stranded stable circular genome (Ma et al., 2019). However, other mitogenome structures have been observed, including circular, linear, branched and numerous small circular molecules (Sloan, 2013). Furthermore, plant mitochondrial genomes (mitogenomes) vary in size even among related species. Most genomes are 200-800 kb in size, and the largest land plant mitogenome is about 11.3 Mb (*Silene conica*) (Sloan et al., 2012). The smallest is only about 66 kb (*Viscum scurruloideum*) (Skippington et al., 2015). Mitogenomes contain abundant repetitive sequences and mediate intergenomic gene transfer, which can lead to variation in genome size and rapid genome rearrangement. Reconstructing the structure of plant mitogenomes is challenging due to their redundant sequences and genomic recombination (Christensen, 2013). Plant mitochondrial markers provide an important complement to plastid DNA markers, indel and minisatellite regions are relatively easy to detect, and are often used to develop markers for breeding and species identification (Sebbenn et al., 2019; Ma et al., 2019). Furthermore, gene sequence transfer between nuclear and organellar genomes is a common phenomenon, as reported in recent studies (Bergthorsson et al., 2003; Li et al., 2022; Ma et al., 2022). As a consequence, the gene content also varies considerably (Wynn and Christensen, 2019).

*P. davidiana* is an excellent germplasm material for the peach breeding research due to its high resistance to many biotic and abiotic stresses. In previous studies, several mitogenomes of *Prunus* species have been sequenced and assembled, such as *P. avium* (Yan et al., 2019) and *P. salicina* (Fang et al., 2021) Although the cp genome of *P. davidiana* has been reported, the mitogenome has not been reported. In the present study, we report the sequencing, assembly and annotation of the *P. davidiana* mitogenome using a combination of the Illumina and PacBio sequencing platforms. In addition, we also analysed repetitive sequences, selective pressures, RNA editing sites and phylogenetic relationships among Rosaceae species in the present study. Gene transfer between nuclear, cp and mitochondrial genomes was also investigated. The results of the present study could improve our understanding of organelle genome evolution in *P. davidiana* and facilitate genomic breeding of *P. davidiana*.

## Materials and methods

### Materials and sequencing

*P. davidiana* plants were grown under natural conditions at the germplasm base of the Jiangsu Academy of Agricultural Sciences (Xuanwu District, Nanjing, China, lat.32^°^02’N, long.118^°^46’E). Total DNA was isolated from fresh leaves using the CTAB method (Varré et al., 2019), and quality was assessed by 1% agarose gel electrophoresis. Qualified samples were sent to Nanjing Jisi Huiyuan Biotechnology Co. (http://www.genepioneer.com/) for Illumina sequencing and Oxford sequencing. Experimental procedures were performed according to a standard protocol (Bi et al., 2020). To obtain the full-length mitochondrial genome with high accuracy. Paired end sequencing (PE) reads (150 bp) were obtained using an Illumina Novaseq 6000 (Illumina, San Diego, CA, USA) and we filtered the original data using fastp (v 0.20.0, https://github.com/OpenGene/fastp) software to obtain high quality clear reads. Long read sequences were obtained from Nanopore PromethION (Nanopore, Oxford, UK) and filtered using filtlong (v0.2.1, https://mirrors.sjtug.sjtu.edu.cn/kali/pool/main/f/filtlong/) software.

### Mitogenome assembly and annotation

Plant mitochondrial genes (CDS and rRNA) are highly conserved. Taking advantage of this feature, reads were mapped to the reference gene sequences (plant 2mitochondrial core genes (https://github.com/xul962464/plant_mt_ref_gene)) using Minimap2 (v2.1) (Ma et al., 2017). Candidate sequences containing multiple core genes and higher alignment quality (covering more complete core genes) were selected as seed sequences. The seed sequences were then compared to the long-read sequencing data with a minimum overlap of 1 kb and at least 70% similarity. Canu (Ye et al., 2017) was used to correct the long-read sequencing data, and bowtie2 (v2.3.5.1) (Cheng et al., 2021) was used to align the short-read sequencing data to the corrected sequence. The preliminary assembly drawing was obtained in Unicycler (Wick et al., 2017), then the final assembly of mtDNA was performed using the long-read sequencing data. Finally, the longest path was selected as the mitochondrial genome of *P. davidiana* on based on the relationship between the linkage and sequence depth.

The structure annotation of the mitochondrial genome was divided into the following steps: (1) The encoding protein and rRNA were aligned with published plant mitochondrial sequences using BLAST and further manual alignment with related species. (2) The tRNA was annotated using tRNAs-canSE (http://lowelab.ucsc.edu/tRNAscan-SE/) with default settings (Goremykin et al., 2012). To obtain more accurate annotation results, the above results were checked and manually corrected using Apollo (Gualberto et al., 2014). The mitochondrial genome was then mapped using OGDRAW (https://chlorobox.mpimp-golm.mpg.de/OGDraw.html).

### Analysis of RSCU and repeat structures

We performed codon preference analysis on the PCGs of the mitogenome and calculated RSCU values using MEGA 7.0 (Xiong et al., 2021). SSRs were detected using the Perl script MISA (http://pgrc.ipkgatersleben.de/misa/) (Liu et al., 2023), with sizes ranging from one to six nucleotides and thresholds of 10, 5, 4, 3, 3 and 3. In addition, REPuter (Clifton et al., 2004) was used to identify the forward, palindromic and tandem repeats.

### Selective pressure analysis

We calculated the non-synonymous (Ka) and synonymous (Ks) substitution rates with PCGs in *P. davidiana*, *M. domestica*, *S. aucuparia*, *R. bibas*, *P. betulifolia* and *F. orientalis*. DnaSP was used to calculate Ka, Ks and Ka/Ks values (Rice et al., 2013).

### RNA editing sites prediction and genome alignments

The *P. davidiana* cp genome (MH460864) was downloaded from the NCBI Organelle Genome Resources Database. The *P. davidiana* mitogenome was searched against the cp genome using BLASTN 2.9.0+ (Zhao et al., 2019). In addition, a BLASTN search (E-value=10^-50^) was performed between the mitogenome and the *P. davidiana* nuclear genome sequence (PRJNA655343). PREP suit (http://prep.unl.edu/) was used to predict the RNA editing sites in the PCGs of the *P. davidiana* mitogenome (the cut-off value was set to 0.2).

### Synteny and phylogenetic tree construction

A pairwise comparison dot plot was generated and the conserved co-linear blocks were plotted. A multiple synteny plot of the *P. davidiana* mitogenome with closely related species was then visualised using MCscanX (Arseneau et al., 2017). A total of 29 complete mitogenomes were downloaded (https://www.ncbi.nlm.nih.gov/) to determine the phylogenetic position of *P. davidiana*. We aligned the 32 common genes of the analysed species in Muscle (Bolger et al., 2014) and constructed the maximum likelihood (ML) phylogenetic tree using MEGA X (1000 bootstrap replicates) (Xiong et al., 2021).

## Results

### Characteristics of the *P. davidiana* mitogenome

**Figure 1:**
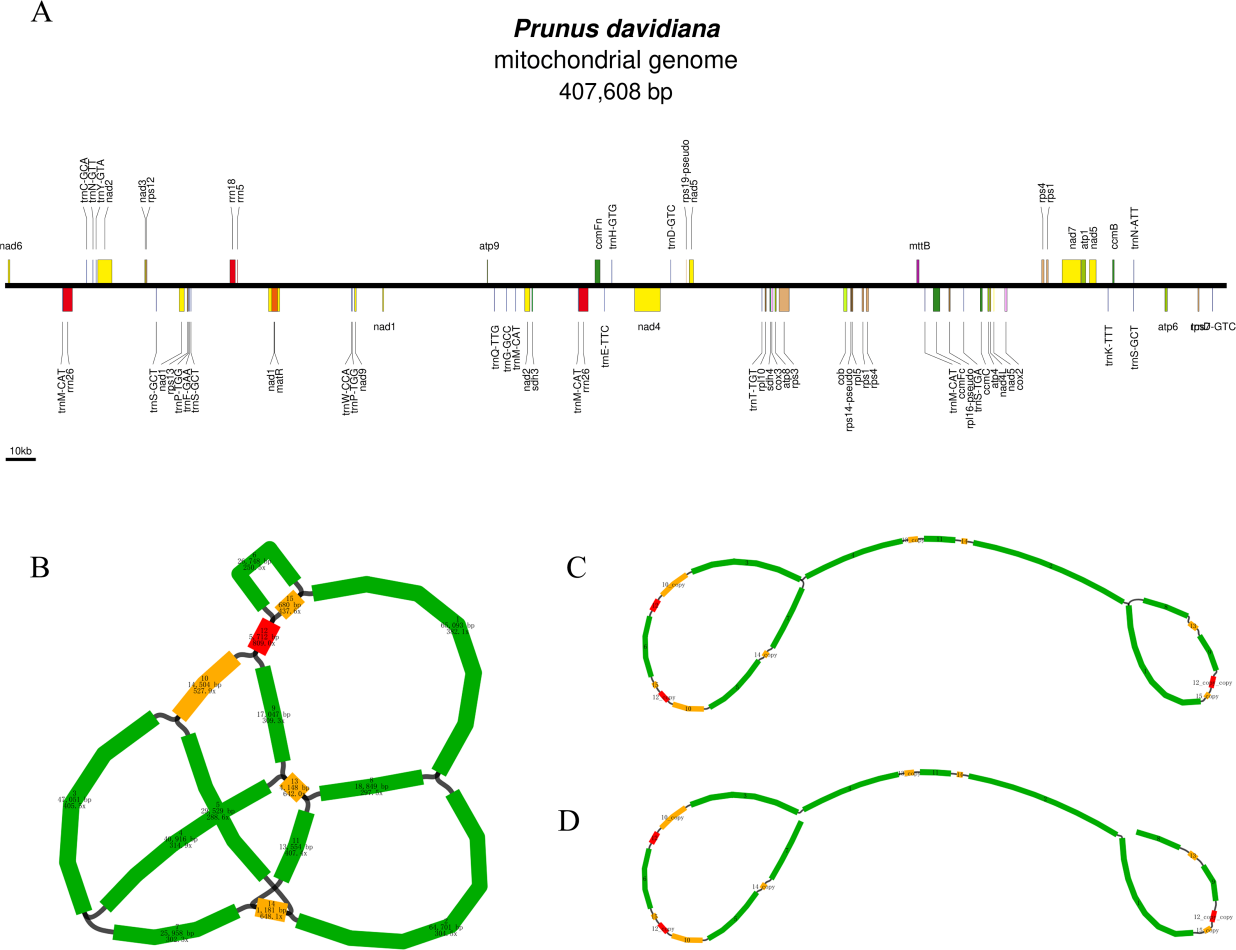
**(A)** The order, orientation and size of gene within the *P. davidiana* mitogenome. Each box is proportional to size of the gene including introns. **(B)** Schematic of mtDNA of *P. davidianas*. **(C)** Unicycler hybrid assembly-based mtDNA of *P. davidianas*. **(D)** The specific solution path is 8-13-9-12-copy-copy-15-copy-1-2-14-11-13-copy-4-3-10-copy-12-6-15-12-copy-10-5-14-copy-7.

The *P. davidiana* mitogenome was sequenced, assembled and annotated. The Oxford Nanopore (9.09 Gb, 22, 312 × coverage, with a read length N50 of ~15.82 kb) and Illumina data (13, 275× coverage) have been submitted in the Sequence Read Archive (SRA) under study accession number SRR25468552 and SRR25468515, respectively (**Supplementary Table S1**). The graph-based mitochondrial genome of *P. davidiana* was assembled, and a specific linear structure was used to represent the entire mitogenome sequence. The total length of the *P. davidiana* mitogenome was 407, 608 bp and the GC content was 45.5% (**Figure 1A**). The genome sequence of *P. davidiana* was submitted to the GenBank database (PQ825745).

A total of 64 genes were annotated in the *P. davidiana* mitogenome, including 39 protein-coding genes (PCGs), 22 tRNA genes and 3 rRNA genes (**Table 1**). Among the 39 PCGs, six contained introns (nad1, nad2, nad4, nad7, ccmFc and rps3). Interestingly, two copy genes (nad1, nad2 and cox1) and one three copy gene (nad5) were found. We also observed five tRNA genes and one rRNA gene located in repeat sequences (trnN-GTT, trnM-CAT, trnP-TGG, trnH-GTG, trnW-CCA and rrn5) (**Figure 1A**).

**Table 1:** The characteristics of genes in *P. davidiana* mitogenome.

Group	Name	Length	Start codon	Stop codon	Amino acids
ATP synthase	*atp1*	1524	ATG	TGA	507
	*atp4*	597	ATG	TAG	198
	*atp6*	774	ACG	TAA	282
	*atp8*	480	ATG	TAA	159
	*atp9*	225	ATG	TGA	74
NADH dehydrogenase	*nad1(2)^a^*	978	ACG	TAA	325
	*nad2(2)^a^*	1467	ATG	TAA	488
	*nad3*	357	ATG	TAA	118
	*nad4^a^*	1488	ATG	TGA	495
	*nad4L*	303	ACT	TAA	100
	*nad5(3)*	2013	ATG	TAA	670
	*nad6*	618	ATG	TAA	205
	*nad7^a^*	1185	ATG	TAG	394
	*nad9*	573	ATG	TAA	190
Cytochrome c biogenesis	*ccmB*	621	ATG	TGA	206
	*ccmC*	753	ATG	TGA	250
	*ccmFc^a^*	1320	ATG	TAG	439
	*ccmFn*	1731	ATG	TGA	576
Maturases	*matR*	1968	ATG	TAG	655
Ubichinol cytochrome c reductase	*cob*	1182	ATG	TGA	393
Cytochrome c oxidase	*cox1(2)*	1566	ATG	TAA	521
	*cox2*	783	ATG	TAA	260
	*cox3*	798	ATG	TGA	265
Transport membrane protein	*mttB*	792	ATA	TAG	264
Ribosomal proteins (LSU)	*rpl5*	555	ATG	TAA	184
	*rpl10*	453	ATG	TAA	150
Ribosomal proteins (SSU)	*rps3^a^*	1650	ATG	TAA	549
	*rps4*	831	ATG	TAA	276
	*rps1*	642	ATG	TAA	213
	*rps7*	447	ATG	TAA	148
	*rps12*	378	ATG	TGA	125
	*rps13*	351	ATG	TGA	116
Succinate dehydrogenase	*sdh3*	324	ATG	TGA	107
	*sdh4*	387	ATG	TGA	128
Transfer RNAs	*trnY-GTA*	83	_	_	_
	*trnN-GTT^b^*	72	_	_	_
	*trnC-GCA*	71	_	_	_
	*trnS-GCT(3)*	65/88	_	_	_
	*trnF-GAA*	74	_	_	_
	*trnK-TTT*	73	_	_	_
	*trnM-CAT(3)*	73/74/77	_	_	_
	*trnP-TGG^b^(2)*	74/75	_	_	_
	*trnE-TTC*	72	_	_	_
	*trnW-CCA^b^*	74	_	_	_
	*trnS-GCT*	65/88	_	_	_
	*trnS-TGA*	87	_	_	_
	*trnD-GTC(2)^b^*	70	_	_	_
	*trnQ-TTG*	72	_	_	_
	*trnG-GCC*	72	_	_	_
	*trnH-GTG^b^*	74	_	_	_
Ribosomal RNAs	*rrn5*	118	_	_	_
	*rrn18*	1863	_	_	_
	*rrn26*	3156	_	_	_

The number of copies are showed after gene names. The genes containing introns and chloroplast-derived genes showed with a and b indicate, respectively.

We visualised the schematic of the mitogenome assembled from the long read data using Bandage software. The schematic contained 14 contigs, the sequence length and sequencing depth of the contigs are shown in **Figure 1B**. The black line indicates the overlapping region between two contigs. These contigs were used to construct a complex multibranched structure. The multiple major branch nodes were resolved using Unicycler software with long reads (Wick et al., 2017). In short, for branch nodes with multiple connections, those connections supported by more long reads are preferred. The genome sequence of a line obtained after solving the branch nodes using Unicycler is shown in **Figure 1B**. The specific solution path is 8-13-9-12-copy-copy-15-copy-1-2-14-11-13-copy-4-3-10-copy-12-6-15-12-copy-10-5-14-copy-7 (**Figures 1C, D**). The plant mitogenome is not unique, and the assembly is not the only form, but is in the midst of dynamic changes, which has facilitated the subsequent analysis.

### Codon usage analysis of PCGs

**Figure 2:**
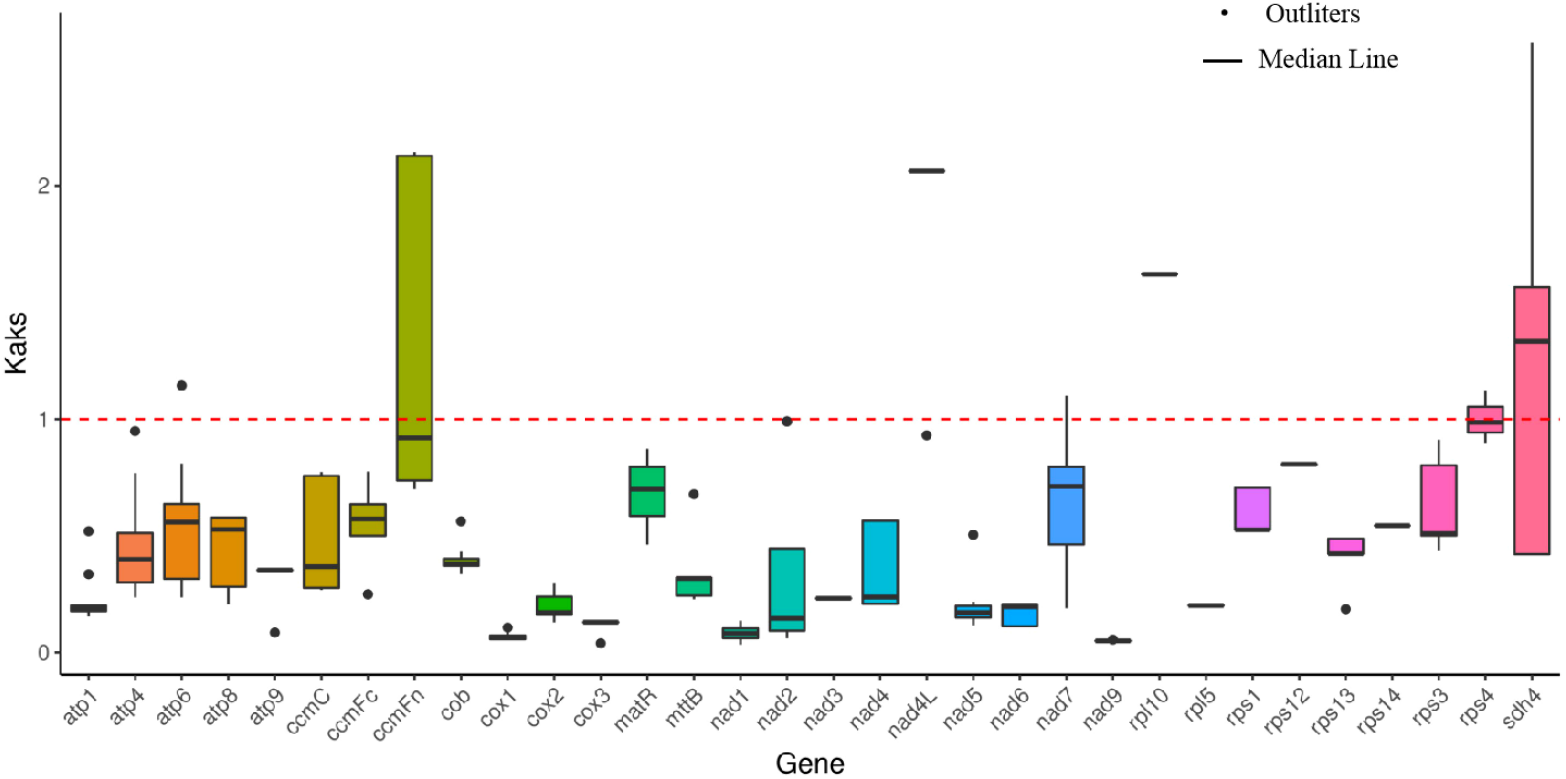
Ka/Ks calculated based on 32 shared genes among the six Rosaceae mitogenome.

Codon usage analysis was performed on 39 PCGs. The codon usage of each amino acid is shown in **Figure 2**. The PCGs in *P. davidiana* had a total length of 30, 234 nucleotides. The typical ATG start codon was found in most PCGs, whereas nad4L and nad1 were ACG, which can be altered by C-to-U RNA editing (**Table 1**). As in other mitogenomes, TAA, TGA and TAG served as stop codons.

**Figure 3:**
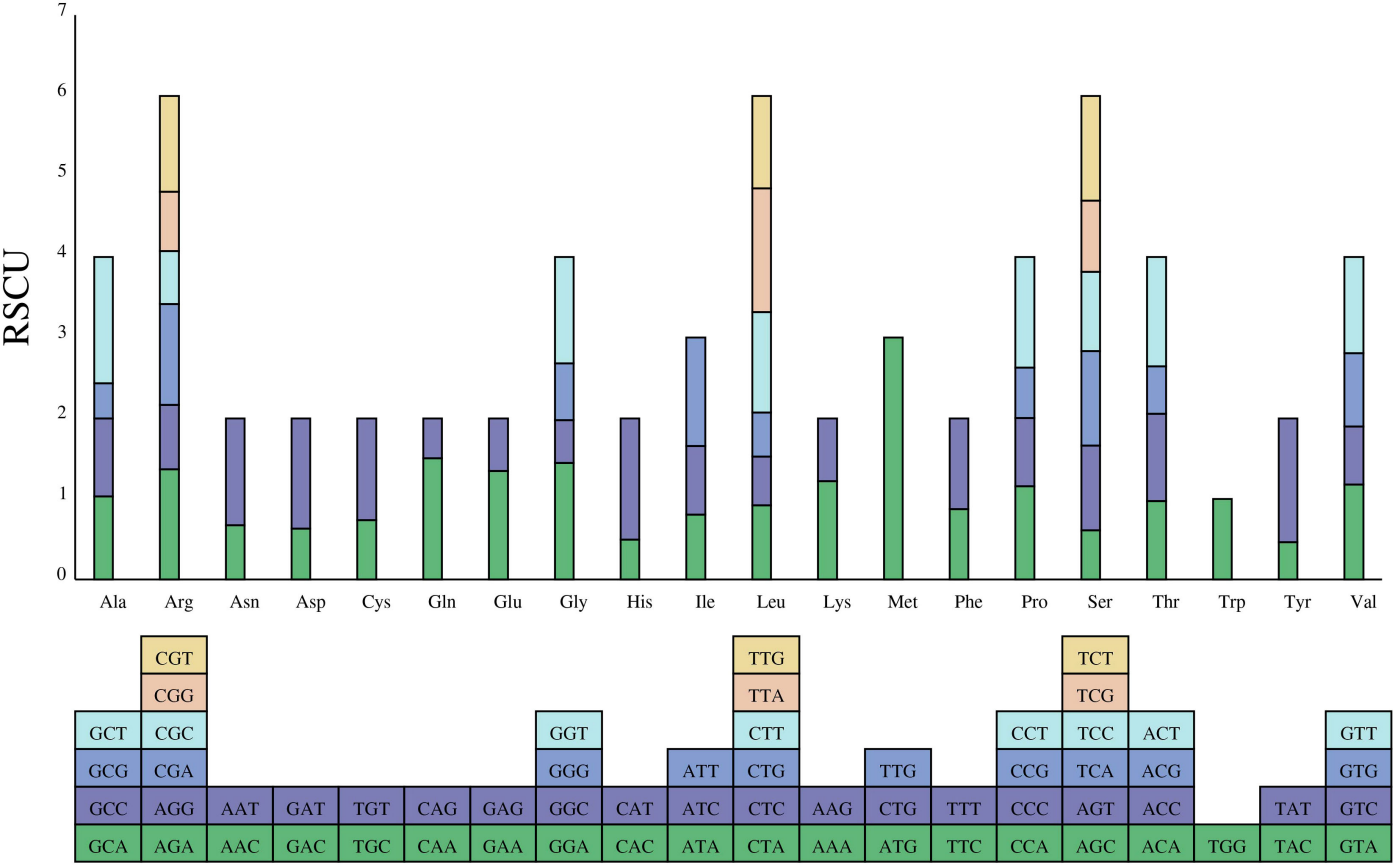
The RSCU analysis in the *P. davidiana* mitogenome. The x-axis was Codon families. RSCU values displayed on bar.

The relative synonymous codon usage (RSCU) of 39 PCGs was also analysed. The 39 PCGs comprised 30, 234 bp encoding 10, 078 codons, excluding termination codons. Codons with RSCU >1 were considered to be preferentially used by amino acids. All RSCU values of NNT and NNA codons, except Ile (ATA), Leu (CTA) and Thr (ACA), were higher than 1.0 (**Figure 3**). The result indicates the existence of a strong As or Ts bias at the third codon position in *P. davidiana* mitogenomes. This phenomenon is also present in other mitogenomes of plant species (Ma et al., 2022).

### Analysis of synonymous and non-synonymous substitution ratios

The non-synonymous to synonymous substitution ratios (Ka/Ks) between any two species among six species (*Malus domestica*, *Sorbus aucuparia*, *Rhaphiolepis bibas*, *Pyrus betulifolia*, *Fragaria orientalis*, and *P. davidiana*) were calculated based on the 32 shared genes in Rosaceae. According to **Figure 2**, the mean values of pairwise Ka/Ks of *ccmFn*, *rps4* and *adh4* were higher than those of other genes. The result suggests that these genes were under positive selection during evolution in the six plant species.

### Prediction of RNA editing sites in PCGs

**Figure 4:**
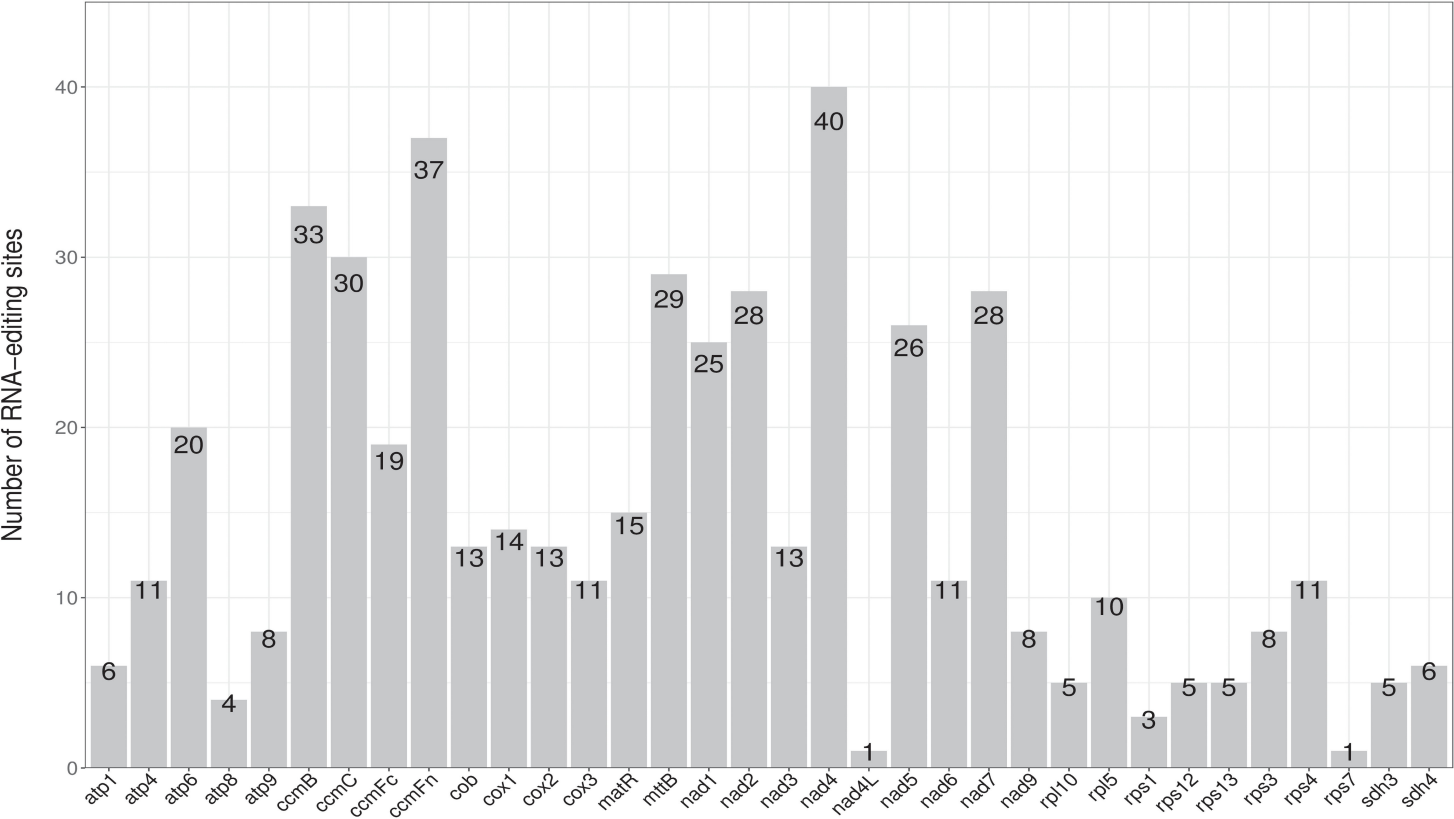
The RNA editing sites distribution in the *P. davidiana* mitogenome. The number shown by grey box represents the RNA editing sites of each gene.

RNA editing events, which are post-transcriptional processes, are enriched in metagenomes (Li, 2018). A total of 502 potential RNA editing sites were identified on 32 mitochondrial PCGs according to predictions from the online PREP suit website (http://prep.unl.edu/) (cut-off value = 0.2). All were found to be C to U base editing. As shown in **Figure 4**, the *nad4* gene encoded the most RNA editing sites (40 sites), whereas *nad4L* and *rps7* encoded only one site. Of the 502 predicted sites, the results were hydrophilic to hydrophobic (13.15%; 66 sites), hydrophobic to hydrophilic (47.01%; 236 sites), hydrophilic to hydrophilic (7.97%; 40 sites), hydrophobic to hydrophobic (31.27%; 157 sites), and hydrophilic to stop (0.60%, 3 sites).

### Analysis of repetitive sequences in the *P. davidiana* mitogenome

Repeated sequences are widespread in the mitochondrial genome and mainly include SSRs, tandem repeats and dispersed repeats (Koren et al., 2017). SSRs are tandemly repeated motifs of one to six bases, that are commonly used as molecular markers in the study and identification of species and the analysis of genetic diversity (Langmead and Salzberg, 2012). In the present study, a total of 132 SSRs were identified in the *P. davidiana* mitogenome, including 56 (38.62%) mono-, 21 (14.48%) di-, 11 (7.59%) tri-, 46 (31.72%) tetra-, 7 (4.83%) penta- and 4 (2.76%) hexanucleotide repeats (**Table 2**). More than 70.34% of the 132 SSRs belonged to monomers and tetramers. In addition, the results showed that 67.86% of the monomers were A/T contents. In previous studies, the proportion of A/T in SSRs contributed to the proportion of A/T in the whole mitogenome (Schattner et al., 2005; Langmead and Salzberg, 2012).

**Table 2:** The SSR motifs frequency in the *P. davidiana* mitogenome.

Motif Type	Number													Total	Proportion (%)
	3	4	5	6	7	8	9	10	11	12	13	15	16		
**Monomer**	**-**	–	–	–	–	–	–	31	6	12	4	1	2	56	38.62
**Dimer**	–	–	17	–	1	1	2	–	–	–	–	–	–	21	14.48
**Trimer**	–	10	1	–	–	–	–	–	–	–	–	–	–	11	7.59
**Tetramer**	44	1	1	–	–	–	–	–	–	–	–	–	–	46	31.72
**Pentamer**	7	–	–	–	–	–	–	–	–	–	–	–	–	7	4.83
**Hexamer**	4	–	–	–	–	–	–	–	–	–	–	–	–	4	2.76
**Total**	55	11	19	0	1	1	2	31	6	12	4	1	2	132	100

The bold values represent the times of repeat.

In addition to the SSRs, 177 forward, 201 palindromic and 24 tandem repeats were detected in the *P. davidiana* mitogenome. The total repeat length was 59, 637 bp, representing 14.63% of the total *P. davidiana* mitogenome. The majority of the forward and palindromic repeats were between 29 and 60 bp, the longest being 20, 216 bp, whereas the tandem repeats were shorter than 39 bp (**Supplementary Table S2**, **Supplementary Table S3**).

### Phylogenetic and syntenic analysis

**Figure 5:**
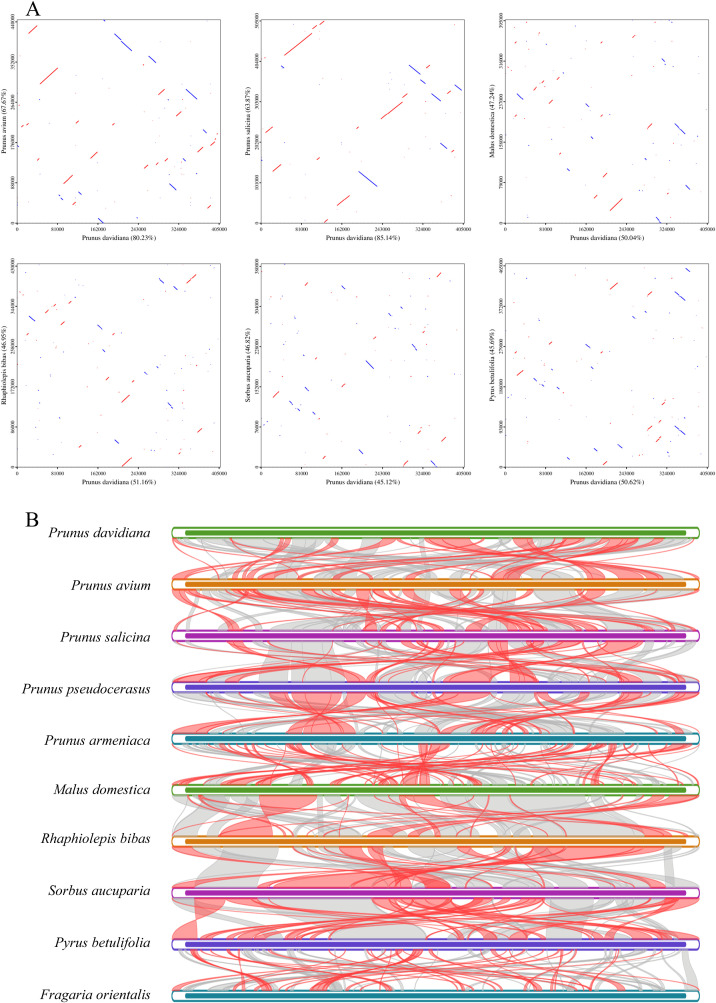
**(A)** Dot-plots analysis of *P. davidiana* with closely related species. **(B)** Synteny analysis of ten mitogenomes. Bars indicated the mitogenomes, and the ribbons showed the homologous sequences between the adjacent species. The common blocks less than 0.5 kb in length are not retained.

The largest co-linear blocks of 35.0 and 34.9 kb were both identified in the dot plot with *P. avium* and *P. salicina*, respectively. In addition, the co-linear blocks were not arranged in the same order, a larger number of homologous co-linear blocks were detected between *P. davidiana* and the closely related species (**Figure 5**). A total of 74 homologous co-linear blocks (>500 bp) were identified between *P. davidiana* and *P. avium*, the largest block being 29,172 bp. Similarly, a total of 85 homologous co-linear blocks (>500 bp) were identified between *P. davidiana* and *P. salicina*, with the largest block being 8, 728 bp in length. The results also showed that the mitogenomes are highly unconserved in structure.

**Figure 6:**
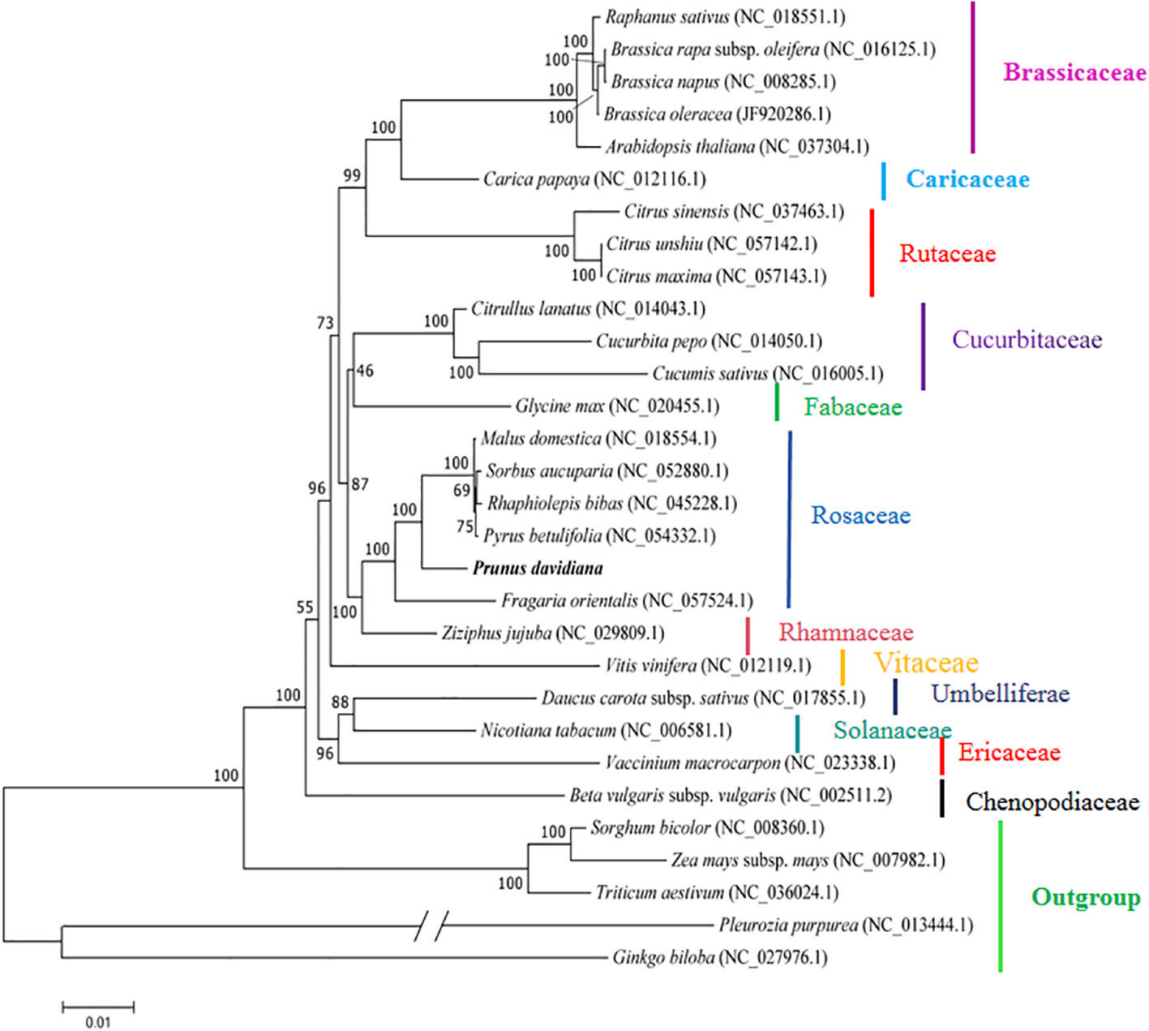
The phylogenetic tree based on 31 single-copy orthologous genes shared among 32 species. The bootstrap support values show at nodes. *Sorghum bicolour*, *Zea mays*, *Triticum aestivum*, *Pleurozia purpurea*, and *Ginkgo biloba* served as outgroups.

The mitogenomes provide an opportunity to confirm the phylogenetic positions of plants. In the present study, to further explore the evolutionary relationships of *P. davidiana* mitochondria, 31 plant mitogenomes were downloaded from the GenBank database (https://www.ncbi.nlm.nih.gov/genome/browse/#!/overview/). The 31 conserved single-copy orthologous genes present in all 32 mitogenomes were selected to construct a phylogenetic tree. Five monocotyledonous species were used as an outgroup. As shown in **Figure 6**, 25 out of 27 nodes in the generated tree had bootstrap support values > 70%, and 17 nodes were 100% supported. The phylogenetic tree strongly supports (bootstrap support = 100%) the close phylogenetic relationship between *P. davidiana* and *P. avium* and *P. salicina* (**Figure 6**). Overall, the results provide a valuable basis for future analyses of the phylogenetic affinities of Rosaceae species.

### Chloroplast and nuclear genes in mitogenome transfer events

DNA fragment transfers are common events in plant evolution between nuclear and organellar genomes. The nuclear and cp genomes of *P. davidiana* were searched using its mitogenome sequences as queries to further understand the characteristics of the sequence transfer event. The 355.99 kb sequences were obtained from the nuclear genome transferred to the mitogenome. In addition, the largest length was 24, 359 bp, and they were mainly between 200 bp and 400 bp. The 27 complete genes (*rps13*, *trnP-TGG*, *trnF-GAA*, *trnS-GCT*, *cob*, *rpl5*, *trnH-GTG*, *trnK-TTT*, *ccmB*, *trnN-ATT*, *ccmC*, *trnD-GTC*, *rps3*, *trnN-GTT*, *rps1*, *trnM-CAT*, *ccmFc*, *trnS-TGA*, *atp4*, *nad4L*, *mttB*, *rps1*, *cox2*, *sdh4*, *cox3*, *atp8* and *rps4*) were included in the common sequences (**Supplementary Table S4**).

The *P. davidiana* mitogenome sequence (407,608 bp) was approximately 2.58 times longer than the cp genome sequence (158,055 bp). The results showed that 16 fragments with a total length of 4, 238 bp had migrated from the cp genome to the mitogenome in *P. davidiana* (**Table 3**). Three of these fragments were longer than 500 bp and the longest was 892 bp. Six intact cp genes (*psaJ*, *petN*, *trnD-GUC*, *trnI-CAU, trnN-GUU*, and *trnM-CAU*) were identified in the fragments. Others were partial sequences of transferred genes. The transferred genes would have facilitated the movement of genetic material throughout *Prunus*.

**Table 3:** Fragments transferred from chloroplasts to mitochondria in *P. davidiana.*.

No.	Identity (%)	Length	Gap opens	CP Start	CP End	Mt Start	Mt End	Gene
1	82.85	548	15	28,856	29,374	251,385	250,841	*petN*
2	83.29	425	16	68,184	68,589	115,536	115,953	*trnW-CCA*; *trnP-UGG*
3	85.63	327	3	66,284	66,604	268,634	268,310	*psbE* (partical)
4	74.10	892	46	139,995	140,858	76,094	75,235	*rrn16* (partical)
5	74.10	892	46	102,892	103,755	75,235	76,094	*rrn16* (partical)
6	85.16	256	9	68,831	69,078	250,715	250,470	*psaJ*
7	89.87	148	3	36,207	36,351	127,344	127,197	*psbC* (partical)
8	81.77	192	6	31,690	31,878	222,236	222,052	*trnD-GUC*
9	96.51	86	1	110,893	110,978	29,356	29,272	*trnN-GUU*
10	96.51	86	1	132,772	132,857	29,272	29,356	*trnN-GUU*
11	96.47	85	0	82	166	202,508	202,424	*trnH-GUG*
12	94.94	79	0	53,938	54,016	170,383	170,305	*trnM-CAU*
13	87.34	79	3	155,493	155,567	325,354	325,276	*trnI-CAU*
14	87.34	79	3	88183	88,257	325,276	325,354	*trnI-CAU*
15	100	31	0	10,543	10,573	128,085	128,115	*atpA* (partical)
16	96.97	33	1	66,248	66,279	268,686	268,654	*psbE* (partical)

### Development of indel markers

**Figure 7:**
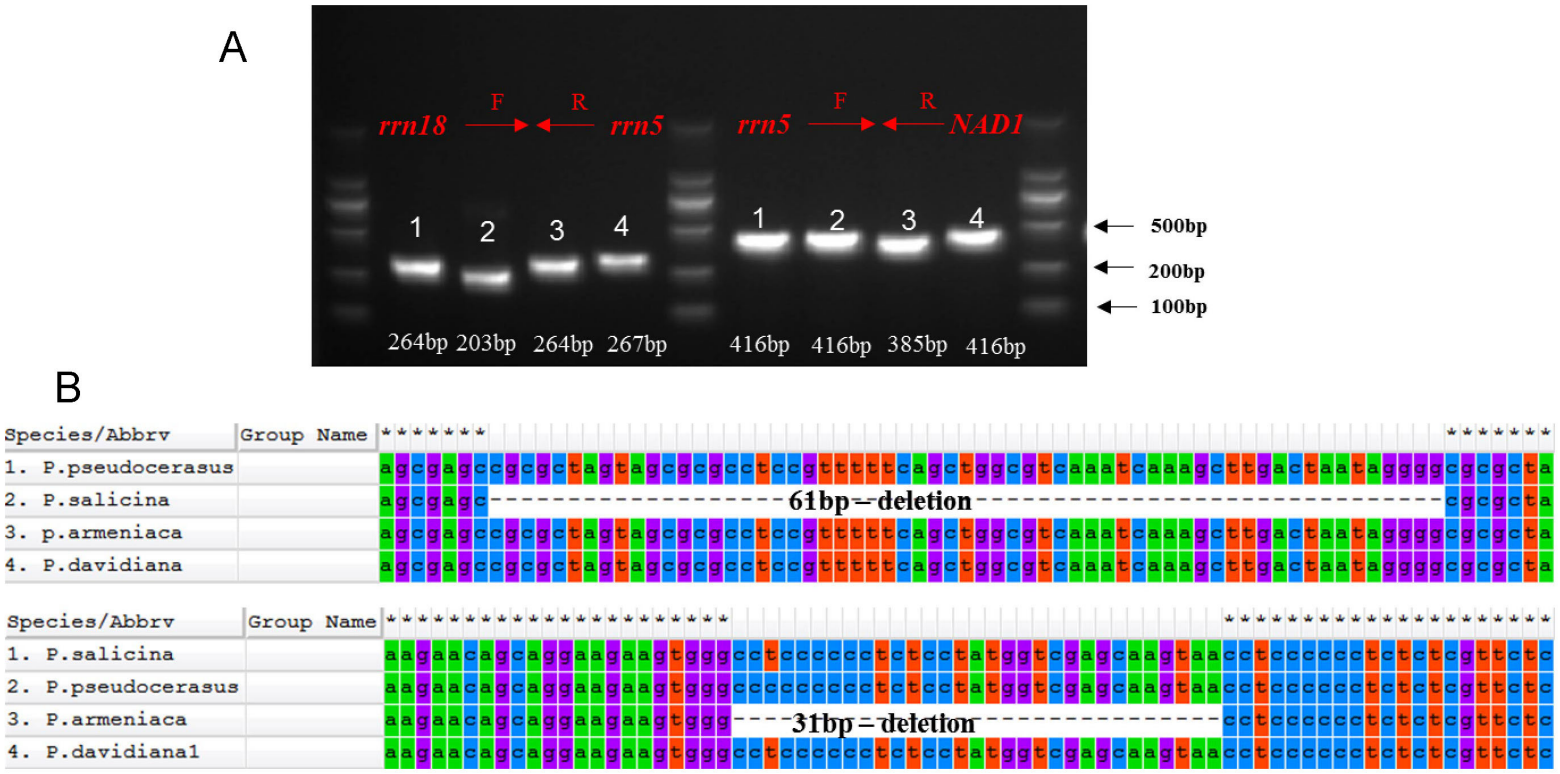
Schematic diagram of the development of the indel markers in four *Prunus* species. **(A)** Results of the PCR amplification of the indel marker in the follow *Prunus* species: *rrn18*-*rrn5* (1) *P. pseudocerasu*s, (2) *P. salicina*, (3) *P. armeniaca*, (4) *P. davidiana*; *rrn5*-*NAD1* (1) *P. salicina*, (2) *P. pseudocerasu*s, (3) *P. armeniaca*, (4) *P. davidiana*; Marker: 2,000 bp ladder. **(B)**: Alignment of the indel markers sequence with MEGA 6.0.

Plant mitochondrial markers are an important complement to the development of plastid DNA markers (Duminil and Besnard, 2021). Indel regions are relatively easy to detect and are often used to develop markers for species identification (Ma et al., 2019). To develop indel markers for *Prunus*, the mitochondrial genome sequences of four *Prunus* species (*P. pseudocerasus*, *P. armeniaca*, *P. salicina* and *P. davidiana*) were aligned, and two detections were detected in the *rrn18-rrn5* (61bp) and *rrn5-Nad1* (31bp) regions. To develop indel markers, the sequence-specific primers were designed and the predicted products were successfully amplified with the conserved regions of flanking *rrn18-rrn5* and *rrn5-Nad1* (**Figure 7A**, **Supplementary Table S5**). The length of the amplified *rrn18*-*rrn5* sequence was similar for *P. pseudocerasus*, *P. armeniaca* and *P. davidiana*. In contrast, the corresponding sequence in *P. salicina* was shorter due to the 61 bp deletion. Similarly, a 31 bp deletion of *rrn5-Nad1* was amplified in *P. armeniaca* (**Figure 7B**). The predicted sizes of the indels were consistent with the sizes of the fragments amplified from the samples analysed in this study. Indel markers are commonly used to distinguish closely related species (Ma et al., 2019; Grosser et al., 2023). However, *Prunus* species have not been identified using this approach.

## Discussion

### Characterisation of the *P. davidiana* mitgenome

Mitochondria are vital organelles in organisms that provide energy for the physiological activities of various cells. Studies have reported that plant mitogenomes are more complex than animal mitogenomes in terms of size variation, repetitive content, protein coding sequences and structure (Lewis et al., 2002; Han et al., 2022). Previous studies have reported the circular, linear, branched and numerous smaller circular molecule structures of mitogenomes (Sloan, 2013). In the present study, we have assembled and characterised the *P. davidiana* mitogenome in detail. The size of the *P. davidiana* mitogenome was 407,608 bp, which is shorter than that of P. salicina (Fang et al., 2021) and P. armeniaca (510, 342 bp; PQ374427), and longer than that of P. pseudocerasus (387, 658 bp; PP968944), similar to the previously reported mitogenome of M. domestica (396, 947 bp) (Kumar et al., 2016). In the previous studies, the gene order and structural features are relatively conservative, while the number genes of P. armeniaca (57) and P. pseudocerasus (55) are less than *P. davidiana* (64) mitgenome. GC content is important for species assessment, and the GC content of *P. davidiana* was 45.5%, which is similar to the typically stable 43%-46% in higher plants (Schattner et al., 2005; Ma et al., 2022). This supports that GC content is highly conserved in higher plants. In the published mitogenomes of Rosaceae, some multiple copy genes have been found, such as atp8, cox2, nad4. Here, a three-copy gene (nad5) was identified, which has hardly been reported before.

### Identification of repeat sequences and RNA editing sites

Repeated sequences are widespread in the mitogenome and include two main categories: tandem and interspersed repeats (Cheng et al., 2021; Bi et al., 2022). They are important for the development of markers for population and evolutionary studies (Kurtz et al., 2001; Thiel et al., 2003; Librado, 2009). In the present study, a total of 59, 637 bp repeats were identified in the *P. davidiana* mitogenome, representing 14.63% of the total *P. davidiana* mitogenome. Such repeats would accelerate intermolecular recombination in mitogenomes during Rosaceae evolution.

In plants, RNA editing plays an important role in protein folding and is enriched in mitochondrial and cp genomes, especially cytidine (C)-to-uridine (U) RNA editing sites (Langmead and Salzberg, 2012; Koren et al., 2017; Sun et al., 2024). Previous studies have identified approximately 491 RNA editing sites in 34 genes in *Oryza sativa* L (Cheng et al., 2021), 486 RNA editing sites in 31 genes in *Phaseolus vulgaris* L (Koren et al., 2017), and 421 RNA editing sites in 26 genes in *Acer truncatum* (Ma et al., 2022). In the present study, 502 RNA editing sites were identified in 32 PCGs in the *P. davidiana* mitogenome. The results showed that the number of RNA editing sites varies greatly between mitogenomes of plant species. Interestingly, all were C-U RNA editing sites. However, the majority of RNA editing sites were associated with cytochrome c biogenesis and NADH dehydrogenase genes, a trend similar to *A. truncatum* (Ma et al., 2022) and *Clematis acerifoli*a (Librado, 2009).

### DNA fragment transfer events

DNA fragment transfer events are common in mitochondria, nuclei and cp of angiosperms. Furthermore, previous studies have suggested that the most prominent direction of transfer is from organellar genomes to nuclear genomes, and then from chloroplast genomes and nuclei to the mitogenomes (Edgar, 2004; Wang et al., 2012; Koren et al., 2017). According to the results of the present study, we found that the 303.0 kb sequences of nuclear DNA were transferred into the 15 molecules in the *P. davidiana* mitogenome. In addition, 16 fragments were identified that were transferred from the cp genome to the mitogenome.

To explore homologous covariance blocks between the related species, the arrangement of homologous genes or sequences was determined using a covariance study. According to the results, the largest co-linear blocks of 35.0 and 34.9 kb were identified in the dot plot with *P. avium* and *P. salicina*, respectively. However, an inconsistent order of co-linear block arrangement was observed. The results suggest that the *P. davidiana* mitogenome has undergone extensive genomic rearrangements.

### Development of indel markers for *Prunus*

Plant plastid genomes are relatively more conserved in gene order or sequence than in intergenic spacers. The intergenic and intron regions tend to be high polymorphism. Many SSRs or indels markers in the mitogenome have been developed to distinguishing plant species (Grosser et al., 2023). In this study, the complete *P. davidiana* mitogenome was assembled and analysed, and two indel loci in the *rrn18-rrn5* and *rrn5-Nad1* (31bp) regions were amplified and analysed. The *Nad1* intergenic region has been commonly developed for markers (Grosser et al., 2023). Although mitogenomes of several *Prunus* species such as *P. pseudocerasus*, *P. armeniaca* and *P. salicina* have been assembled, the molecular markers were not reported in previous studies. Therefore, the two developed indel markers may be applicable for species classification and identification of *Prunus* species.

## Conclusions

In this study, we sequenced, assembled and annotated the mitogenome of *P. davidiana*. Detailed analyses of the mitogenome were performed based on DNA and amino acid sequences. The total length of the *P. davidiana* mitogenome is 375,671 bp, including 15 linear molecules, with a GC content of 45.5%. We annotated 64 genes, including 39 PCGs, 22 tRNA genes and 3 rRNA genes. Codon usage, repetitive sequences, Ka/Ks, RNA editing, synteny and DNA fragment transfer events were also analysed. To confirm the evolutionary status, we performed a phylogenetic analysis of the *P. davidiana* mitogenome and 31 other plants. We also developed two indel markers for *Prunus*. The results of this study provide valuable information and a theoretical basis for future *P. davidiana* research and breeding activities.

## Data Availability

The datasets presented in this study can be found in online repositories. The names of the repository/repositories and accession number(s) can be found in the article/**Supplementary Material**.
